# Microenvironmental Geometry Guides Platelet Adhesion and Spreading: A Quantitative Analysis at the Single Cell Level

**DOI:** 10.1371/journal.pone.0026437

**Published:** 2011-10-20

**Authors:** Ashley Kita, Yumiko Sakurai, David R. Myers, Ross Rounsevell, James N. Huang, Tae Joon Seok, Kyoungsik Yu, Ming C. Wu, Daniel A. Fletcher, Wilbur A. Lam

**Affiliations:** 1 Division of Pediatric Hematology/Oncology, Department of Pediatrics, Emory University School of Medicine, Atlanta, Georgia, United States of America; 2 Wallace H. Coulter Department of Biomedical Engineering, Georgia Institute of Technology, Atlanta, Georgia, United States of America; 3 Winship Cancer Center, Emory University School of Medicine, Atlanta, Georgia, United States of America; 4 Department of Bioengineering, University of California, Berkeley, California, United States of America; 5 Division of Pediatric Hematology/Oncology, Department of Pediatrics, University of California San Francisco, San Francisco, California, United States of America; 6 Department of Electrical Engineering and Computer Science, University of California, Berkeley, California, United States of America; 7 Department of Electrical Engineering, Korea Advanced Institute of Science and Technology, Daejeon, Republic of Korea; 8 Graduate Group in Biophysics, University of California, Berkeley, California, United States of America; Swiss Federal Institute of Technology Zurich, Switzerland

## Abstract

To activate clot formation and maintain hemostasis, platelets adhere and spread onto sites of vascular injury. Although this process is well-characterized biochemically, how the physical and spatial cues in the microenvironment affect platelet adhesion and spreading remain unclear. In this study, we applied deep UV photolithography and protein micro/nanostamping to quantitatively investigate and characterize the spatial guidance of platelet spreading at the single cell level and with nanoscale resolution. Platelets adhered to and spread only onto micropatterned collagen or fibrinogen surfaces and followed the microenvironmental geometry with high fidelity and with single micron precision. Using micropatterned lines of different widths, we determined that platelets are able to conform to micropatterned stripes as thin as 0.6 µm and adopt a maximum aspect ratio of 19 on those protein patterns. Interestingly, platelets were also able to span and spread over non-patterned regions of up to 5 µm, a length consistent with that of maximally extended filopodia. This process appears to be mediated by platelet filopodia that are sensitive to spatial cues. Finally, we observed that microenvironmental geometry directly affects platelet biology, such as the spatial organization and distribution of the platelet actin cytoskeleton. Our data demonstrate that platelet spreading is a finely-tuned and spatially-guided process in which spatial cues directly influence the biological aspects of how clot formation is regulated.

## Introduction

At sites of vascular injury, platelets initiate the formation of the hemostatic plug. Decades of research have characterized the receptors/ligands for platelet adhesion and activation and the underlying biochemical signaling that govern these processes [1], [2]. In addition, further work has shown that the molecular machinery for platelet adhesion and spreading is strikingly similar to those in other motile cells [3]. Previous studies have shown that these nucleated mammalian cells sense the physical properties, such as geometry and rigidity, of the local environment, and transduce these mechanical signals to regulate cell morphology and spreading [4].

Microcontact printing techniques have recently been applied to platelet adhesion [5], [6], but there currently is no published data that systemically characterizes and quantifies, at the single platelet level, how the microenvironmental geometry influences platelet spreading, and what the physical limitations and constraints are. This concept of physical and microenvironmental control of platelet function is important to our understanding of platelet physiology and clot formation. Indeed, as fibrin formation takes place on the surface of activated platelets, investigation into the spatial regulation of platelet spreading will provide valuable insight into how clot formation itself is regulated and may have implications in bleeding and thrombotic disorders [7]. To quantitatively investigate how single platelets guide spreading based on the geometry of their microenvironment, we combined standard platelet adhesion techniques with deep UV photolithography and micro/nanocontact protein printing using fibrinogen and collagen, which mediate platelet adhesion at sites of vascular injury [8], [9].

## Materials and Methods

### Platelet isolation and preparation

3 mL of blood were drawn from healthy adults by venipuncture into ACD (Becton-Dickinson). This protocol was approved by University of California, Berkeley Emory University IRBs and written informed consent was received from all participants. Platelets were isolated as previously described [10]. Platelets were then resuspended in HEPES-Tyrode buffer with 5 mg/mL bovine serum albumin (BSA) and labeled with a constitutively fluorescent membrane dye (CellMask, Invitrogen). Platelet concentration was then diluted to 2×10^7^ platelets/mL in HEPES-Tyrode with BSA.

### Protein Micro/nanocontact Printing

To enable submicron resolution of single platelet adhesion and spreading, we used deep UV photolithography to create micro/nanofabricated features on silicon chips to serve as protein stamping molds (Supporting Information S1). Micropatterns of Type I collagen-FITC (Exalpha) or fibrinogen-Alexa 488 (Invitrogen) on glass coverslips were created using a microcontact printing methodology previously described (Supporting Information S1) [11]. Platelets were then incubated on the protein micro/nanopattern for 1 to 2 hours and monitored using fluorescence confocal microscopy. For the actin and tubulin experiments, non-stained platelets were fixed with 1% formaldehyde after incubation on the protein micropattern and permeablized. Actin filaments and β1-tubulin were then stained with Alexa Fluor 555 phalloidin (Invitrogen) and mouse monoclonal antibody against β1-tubulin (Abcam) followed by Alexa Fluor 488 goat antibody against mouse IgG (Invitrogen), respectively. Images were obtained using an epifluorescence microscope (Nikon Eclipse Ti2000-U) with 40× and 60× objectives.

## Results

### Quantitatively Analysis of how Microenvironmental Geometry Guides Platelet Spreading

**Figure 1:**
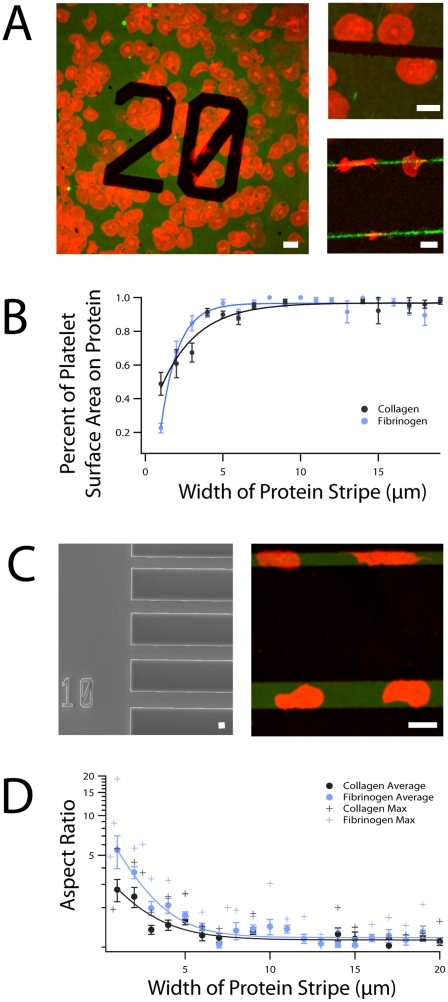
Spatial regulation of platelet spreading. (**A**) Platelets (red) spread on either collagen or fibrinogen-adsorbed micropatterns (green) on glass surfaces and follow the geometric boundaries of the protein micropattern with high fidelity. Non-patterned areas of glass are blocked with BSA and appear dark. (Left panel) Platelets spread along the edges of an arbitrary geometric protein micropattern. (Upper right) Platelets spread and conform to the boundaries of a wider micropatterned protein “stripe”. (Lower right) On thinner micropatterns, the fidelity of platelet spreading along the micropattern boundaries is decreased. (**B**) Systematic variation in protein micropatterned stripe width reveals the physical limitation of geometry-guided platelet spreading. On stripes of protein thinner than 5–6 µm, only a fraction of a platelet's final spread area is on protein. This is contrasted with wider protein stripes where almost all platelets spread entirely on protein. As protein width increases, the total percentage of platelet area spread on protein approaches 1. Bullet points (•) denote average data and fitted lines denote exponential fits to average data. Error bars shown denote standard error of the mean. (**C**) (Left) An electron micrograph of the microfabricated chip used as a microstamp mold for protein micropatterning. The chip is comprised of “plateaus” of the same height but varying width. Microstamps using this mold have protein stripes varying in width from 0.5 to 20 µm and separated by a constant 10 µm. The “10” is a fiduciary marker indicating the stamped protein micropattern width in micrometers. (Right) Platelets that spread on thin protein stripes are observed to have a high aspect ratio. (**D**) As protein micropattern width increases, aspect ratio of the spread platelets approaches 1. On thinner micropatterned protein stripes, platelet spreading was more constrained and higher platelet aspect ratios were observed, with a maximum of 19.0. Bullet points (•) denote average data and fitted lines denote exponential fits to average data. Error bars shown denote standard error of the mean, and crosses (+) denote the maximum aspect ratios observed at a given protein width. Scale bars on all images denote 5 µm. N = 105 and 179 platelets on collagen and fibrinogen, respectively.

In a standard adhesion assay, platelets adhere to protein-coated (e.g. collagen or fibrinogen), BSA-blocked glass surfaces and spread in all directions [12], [13]. To investigate the spatial guidance of platelet spreading, we combined the standard platelet adhesion assay with micro/nanocontact protein printing. This technique enables the “stamping” of micro-to-nanoscale protein patterns onto glass surfaces with high precision. Upon contact and adhesion with fluorescently-labeled fibrinogen or collagen micropatterns on the glass surface, platelets extended filopodia in multiple directions. Platelet spreading ensued but was restricted by the geometric constraints of micropatterns with >5 µm features (Figure 1A (left panel), Supporting Information S1). At the interfaces between those micropatterns and blocked glass, platelets spread only onto the microstamped protein. As platelet spreading continued, the platelets followed the geometric boundaries of the micropatterns with high fidelity and took on the morphology of the protein micropatterns themselves. These results are similar to those reported for motile nucleated mammalian cells, such as fibroblasts, using similar microstamping techniques [4].

To quantify the limitations of geometry-guided spreading of single platelets, the widths of the micropatterned protein “stripes” were systematically varied. Platelets spread to the pattern edges and conformed to the geometric morphology of wider micro/nanopatterns (Figure 1A, (upper right panel)). As stripe width decreased, the fidelity of platelet spreading corresponding to the boundaries of the patterned surface decreased and instead nonspecifically spread onto the BSA-blocked glass (Figure 1A, (lower right panel)). Systematic variation of micro/nanopattern width revealed that above stripe widths of 5 µm, platelet spreading occurred almost exclusively on the protein micro/nanopattern (Figure 1B, Supporting Information S1). Altering the protein concentration thereby increasing the protein ligand density of the micropattern did not reveal any differences in the fidelity of spreading.

To determine how the shapes of individual platelets were affected by geometric guidance, the platelet aspect ratios, or the ratio of a platelet's greatest length along the protein to its greatest width perpendicular to the protein stripe (Figure 1C (left panel) and Figure 1C (right panel)), were calculated for platelets on stripes of varying widths. For protein stripes 6 µm wide and above, the platelet aspect ratio approached 1 as platelets spread in all directions equally. On thinner micropatterned protein stripes, platelet spreading was more constrained and higher platelet aspect ratios were observed, with a maximum of 19.0 (Figure 1D, Supporting Information S1, Video S1). Platelets were able to conform to micropatterned stripes as thin as 0.6 µm. These experiments suggest that while the geometry of the microenvironment regulates platelet spreading, physical limits exist as to how much platelets can conform to the morphology of the micropattern. No difference in platelet spreading or aspect ratio was detected between fibrinogen and collagen micropatterns or at different concentrations of stamped fibrinogen or collagen.

### Platelets span <5 µm gaps between protein micropatterns

**Figure 2:**
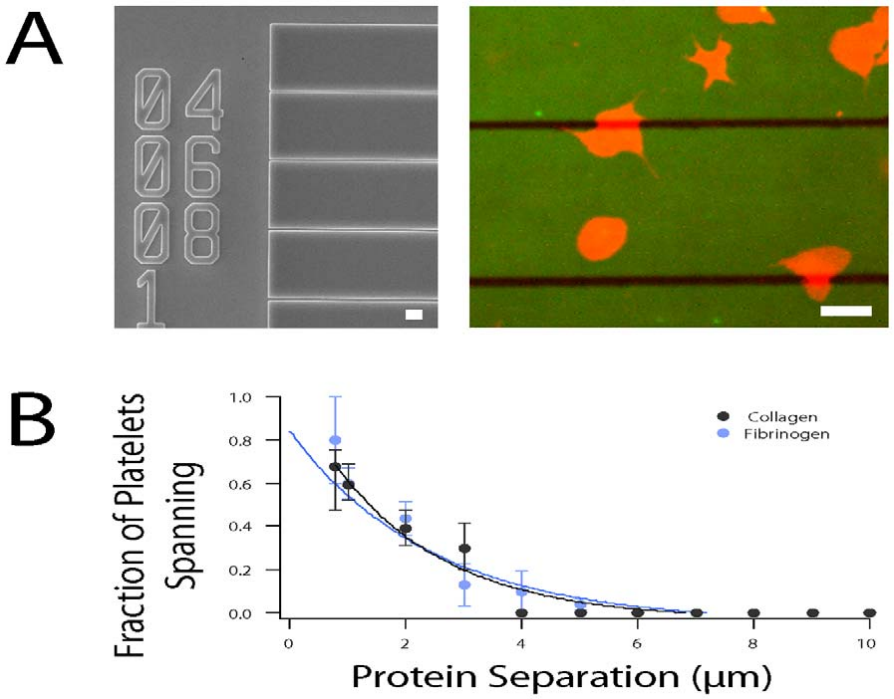
Platelets span gaps between protein micropatterns. (**A**) (Left) An electron micrograph of the microfabricated chip used as a microstamp mold. Microstamps using this mold have regions where constant 10 µm bands of protein micropatterns are separated by areas of BSA-blocked, non-patterned glass ranging from 0.5 to 20 µm in width. The numbers are fiduciary markers indicating separation in micrometers. (**A**) (Right) At protein separations of less than 5 µm, platelets spanned gaps in protein separation and spread on both surfaces. (**B**) The fraction of total platelets in contact with an edge that spread across protein separations decreases as separation increases. At separations larger than 5 µm no platelets are observed to span. Scale bars denote 5 µm. Error bars represent standard error of the mean. Bullet points (•) denote average data and lines (-) denote exponential fits. N = 134 and 196 platelets for collagen and fibrinogen, respectively.

Occasionally, platelets were observed to span and spread over small gaps of non-micropatterned, BSA-blocked glass. To quantitatively investigate this phenomenon, we created protein stripes of constant width with increasing separations between 0.5 and 20 µm and observed platelets spanning the micropattern boundaries (Figure 2A (left panel) and Figure 2A (right panel)). When protein stripe separation was 4–5 µm or less, platelets were able to span and spread over those gaps (Figure 2B). Above that separation distance, platelets could not span the stripes, and spreading was confined to the protein micropattern (Video S2). Interestingly, as the largest separation spanned by platelets on both collagen and fibrinogen is between 4–5 µm, which approximates platelet filopodia length (3.10±0.8 µm, mean±SD, n = 28), it is possible that the limit of filopodial extension also determines limit of platelet spanning.

### Platelets use filopodia to sense the geometry of their microenvironment

**Figure 3:**
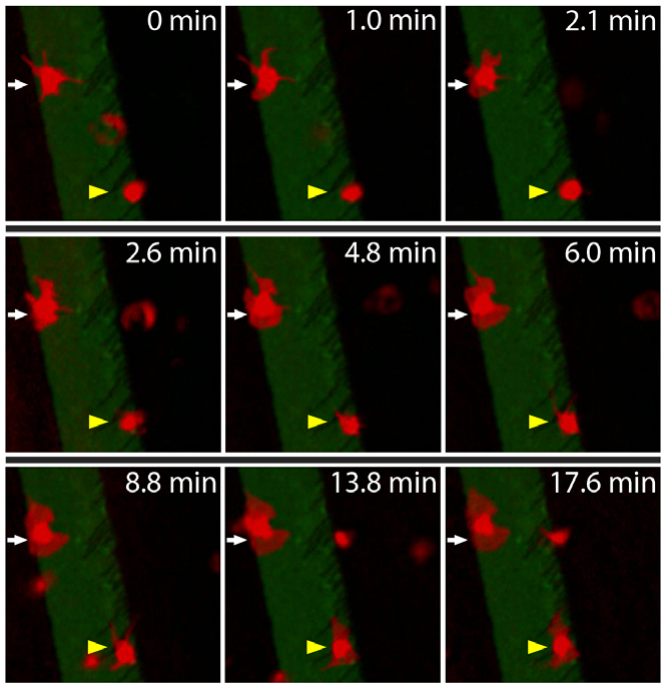
Platelets use filopodia to sense the geometry of their microenvironment and guide spreading. This time lapse sequence focuses on two fluorescently stained platelets (red), which have come into contact with the micropatterned collagen (green). At time 0, one platelet (white arrow) has already adhered to the collagen microstamp, and has extended filopodia. The filopodia dynamically extend in all directions, touching a number of different areas around the platelet. Filopodia will then either retract or firmly attach to the microstamp. Over the course of several minutes, lamellipodia then fill in the areas between the filopodia, but are constrained by the microstamp geometry. Starting at 4.8 minutes another platelet (yellow triangle), comes into contact with the micropatterned collagen and adheres. Filopodia appear to sense the microenvironment by extending and dynamically moving around the platelet until they either retract or come into contact with and attach to the collagen micropattern. As platelet spreading then ensues, lamellipodia fill in the space between the filopodia while conforming to the geometric constraints of the collagen micropattern. Other platelets in suspension occasionally appear but do not come into contact with the collagen micropattern and instead pass through the field of view.

To investigate the underlying mechanisms of how platelets sense microenvironmental geometry, time lapse fluorescence videomicroscopy of platelets interacting with the boundaries of protein microstamps were acquired. Upon contact with non-patterned, BSA-blocked glass, platelets extended and retracted filopodia in multiple directions (Figure 3, Video S3, and Video S4). When the protein micropatterns were within the span of extended filopodia, platelets moved onto the micropatterns and spread onto the protein surface along those filopodia, conforming to the micropattern boundaries (Video S1). Platelets with filopodia that did not come into contact with protein micropatterns continued to extend and retract filopodia but did not spread for the duration of the experiment. Therefore, these experiments suggest that platelet filopodia are involved in probing and “sensing” the geometry of their microenvironment to guide spreading, which is consistent with how other motile cells use filopodia for sensing [14].

### Microenvironmental geometry affects the organization and spatial distribution of the platelet cytoskeleton

**Figure 4:**
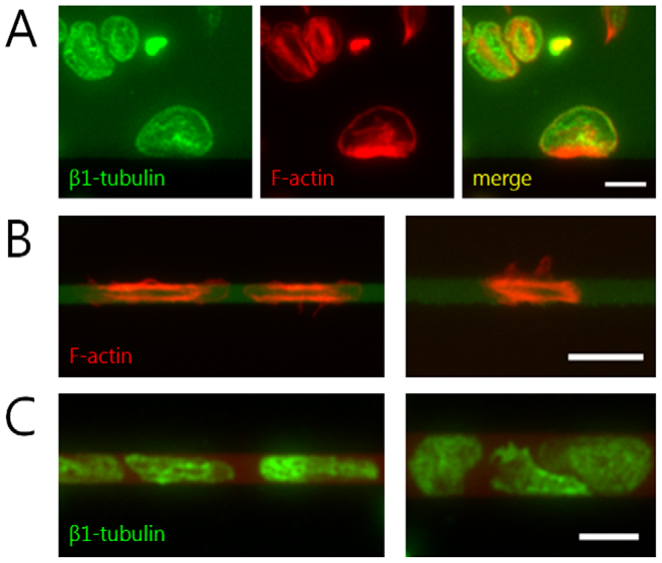
Microenvironmental geometry affects the organization and spatial distribution of the platelet cytoskeleton. (**A**) A platelet geometrically constrained by a fibrinogen micropattern (dull green) exhibits distinct differences between the spatial distribution of polymerized actin (red) and β1-tubulin (bright green). Whereas the density of actin filaments is highest at the platelet edge at the micropattern boundary, β1-tubulin is more homogenously distributed throughout the platelet. (**B**) In platelets confined by thinner micropatterns, large amounts of filamentous actin (red) are distributed at both boundaries of the micropatterned fibrinogen (left) and collagen (right) stripes. (**C**) In contrast, the distribution of β1-tubulin (green) is not affected by the geometric constraints of the fibrinogen micropattern (red). Actin filaments and β1-tubulin were visualized via fluorescent phalloidin and immunofluorescence, respectively. Scale bars on all images denote 5 µm.

To investigate how the spatial cues of the microenvironment may directly influence platelet biology, we stained platelets that were geometrically constrained on collagen and fibrinogen micropatterns with fluorescent phalloidin, which binds to polymerized filamentous actin, and anti-β1-tubulin. Interestingly, we observed that geometrical constraint of platelets on micropatterns lead to significant alterations in the cytoskeletal architecture (Figure 4A, B, C). Specifically, we found that the density of actin filaments is much higher at the edge of the platelet at the micropattern/BSA boundary than elsewhere in the platelet, providing evidence that geometric constraint does in fact influence platelet biology. In contrast, we found that geometric constraint of the platelet does not affect the spatial distribution of tubulin. These experiments suggest that the spatial cues of the microenvironment affect specific and distinct biological pathways within the platelet.

## Discussion

Our study of platelet spreading on protein micro/nanopatterns shows that platelets regulate their spreading and morphology in response to geometrical constraints of the microenvironment, which is consistent with recent research [5]. Using protein stripes of defined widths, we then quantitatively determined the limits of geometry-guided platelet spreading at the single cell level. For physical features greater than 5 µm, platelets conform to the microenvironmental geometry with high fidelity (Figure 1). Below 5 µm, this spreading fidelity decreases with feature size, likely due to the mass effect of the platelet itself. In addition, as platelets extend filopodia, they are observed to “bridge” and spread over non-protein patterned areas of up to 5 µm in distance, consistent with the span of maximally extended filopodia. Finally, we show that the microenvironmental geometry directly affects platelet biology, such as the spatial organization and distribution of the platelet actin cytoskeleton.

These findings have implications for our understanding of hemostasis and introduce the concept of spatial regulation of clot formation. Platelet recruitment to the site of vascular injury is the first step in hemostasis. Our data suggest that the adherent platelets then spread onto those injured sites with high specificity and submicron spatial resolution and precision. As the platelets adhere to collagen and activate, they expose procoagulant phosphatidylserine, simulating assembly of the tenase and prothrombinase complexes [15]. Thus, the activated platelet surface acts as a physical substrate for the propagation phase of coagulation, and spatial regulation of platelet spreading potentially affects the spatial and physical boundaries of overall clot formation itself. Furthermore, as fibrinogen mediates platelet-platelet binding in a growing thrombus, our fibrinogen data suggest that platelet aggregation itself may be spatially regulated as well. Biochemically, hemostasis is tightly controlled with multiple feedback mechanisms. Our observations suggest that this process is also highly spatially controlled, thereby potentially providing another mechanism by which hemostatic plug formation occurs only at sites of vascular injury. The capability of platelets to span <5 µm distances may imply that spatial guidance is less important for micron-scale, subclinical sites of vascular injury. In addition, our data show that the microenvironmental geometry directly affects biological processes such as the reorganization of the platelet cytoskeleton. Further studies, with and without the integration of flow, are needed to investigate how spatial cues affect other biological pathways involving platelet activation such as integrin activation, granule release, calcium signaling, and phosphatidylserine exposure. Only then will we be able to fully comprehend the implications of how the spatial aspects of the microenvironment regulate platelet physiology and clot formation.

## Supporting Information

Supporting Information S1 Supporting Information regarding Microcontact Printing, Supporting Information regarding. Supporting Figure 1 . Schematic of microfabrication. In this process, deep UV (DUV) lithography, plasma-enhanced chemical vapor deposition (PECVD), and reactive ion etching (RIE) were used. PR = photoresist. Supporting Information regarding Electron Micrograph Acquisition. Supporting Information regarding Data Analysis. Supporting Figure 2 . Images of platelets conforming to boundaries of the protein micropattern. Platelets are stained with CellMask (Invitrogen), a constitutive fluourecent membrane dye, and appear red. The micropatterns are comprised of fibrinogen-Alexa 488 (Invitrogen) and appear green. Scale bar denotes 5 µm. Supporting Figure 3 . Additional image showing platelets conforming to the boundaries of the protein micropattern and adjust their spreading accordingly. Scale bar denotes 5 µm. Supporting Figure 4 . On thinner micropatterned protein stripes (<1 µm), platelet spreading was more physically constrained and higher platelet aspect ratios were observed, with a maximum of 19. Scale bar denotes 5 µm.
(DOC)

Video S1 **Geometrically constrained platelet spreading.** One platelet (left) lands on a BSA-blocked glass region near a narrow collagen micropatterned stripe. It then extends filopodia and moves onto the micropattern. While spreading, the platelet conforms to the boundaries of the protein and assumes a rectangular morphology. Meanwhile, another platelet (upper right) lands on a thinner protein stripe and exhibits similar behavior.
(WMV)

Video S2 **Platelet behavior at the protein-glass interface.** A platelet drifting past a fibrinogen micropattern lands on a region of BSA-blocked glass adjacent to the protein pattern. The platelet then extends filopodia and crawls over onto the protein region before fully spreading. As the distance of the non-patterned regions was >5 µm, the platelet could not span the micropatterned stripes, and spreading was confined to the protein micropattern. Elapsed time: 15 minutes, 50 seconds.
(WMV)

Video S3 **Platelet sensing using filopodia.** Time lapsed responses of platelets as they come into contact with a microstamped protein surface. The platelets extend filopodia, which dynamically move around and either anchor to the protein or retract when not in contact with the protein. Lamellipodia then fill in the area between filopodia, and stay within the microstamped boundary. Elapsed Time: 22 minutes, 20 seconds.
(WMV)

Video S4 **Platelet sensing using filopodia.** Time lapsed responses of platelets as they come into contact with a microstamped protein surface. The platelets extend filopodia, which dynamically move around and either anchor to the protein or retract when not in contact with the protein. Lamellipodia then fill in the area between filopodia, and stay within the microstamped boundary. Elapsed Time: 3 minutes, 10 seconds.
(WMV)
