# Supplementary material for: Microenvironmental Geometry Guides Platelet Adhesion and Spreading: A Quantitative Analysis at the Single Cell Level
Source: PLoS One. 2011 Oct 20;6(10):e26437. doi: 10.1371/journal.pone.0026437 (PMC3197646; doi:10.1371/journal.pone.0026437)
Supplement: Supporting Information S1 — Supporting Information regarding Microcontact Printing, Supporting Information regarding. Supporting Figure 1 . Schematic of microfabrication. In this process, deep UV (DUV) lithography, plasma-enhanced chemical vapor deposition (PECVD), and reactive ion etching (RIE) were used. PR = photoresist. Supporting Information regarding Electron Micrograph Acquisition. Supporting Information regarding Data Analysis. Supporting Figure 2 . Images of platelets conforming to boundaries of the protein micropattern. Platelets are stained with CellMask (Invitrogen), a constitutive fluourecent membrane dye, and appear red. The micropatterns are comprised of fibrinogen-Alexa 488 (Invitrogen) and appear green. Scale bar denotes 5 µm. Supporting Figure 3 . Additional image showing platelets conforming to the boundaries of the protein micropattern and adjust their spreading accordingly. Scale bar denotes 5 µm. Supporting Figure 4 . On thinner micropatterned protein stripes (<1 µm), platelet spreading was more physically constrained and higher platelet aspect ratios were observed, with a maximum of 19. Scale bar denotes 5 µm. (DOC) [file pone.0026437.s001.doc]

**Supporting Information**

*Microcontact Printing*

A silicon chip with precise microfabricated features was used as a mold for protein microcontact printing. Polydimethylsulfoxide (PDMS) was then cast and cured overnight at 60^0^C. 50 μL of 0.1 mg/mL Type I bovine collagen-FITC or 0.1-0.5 mg/mL fibrinogen-Alexa 488 was incubated on the surface of each PDMS stamp overnight in a humidified environment. The stamp was then washed with purified water, air-dried with pressurized nitrogen gas, and placed protein-side face down onto a clean coverslip where it was allowed to sit for 10-15 minutes. The PDMS stamp was removed and a 10 mg/mL solution of BSA in phosphate buffered saline (PBS) was used to block the exposed glass around the stamped region for an hour. The BSA solution was then washed off the micropatterned glass surface and 200 μL of the platelet suspension was added. Final concentrations of 1 mM CaCl_2_  or 1 mM MgCl_2_ was added to the platelet suspension for fibrinogen and collagen microstamp experiments, respectively, as previously described^1,2^. Platelets were then incubated on the micropatterned protein for 1 to 2 hours and monitored using fluorescence confocal microscopy.

###
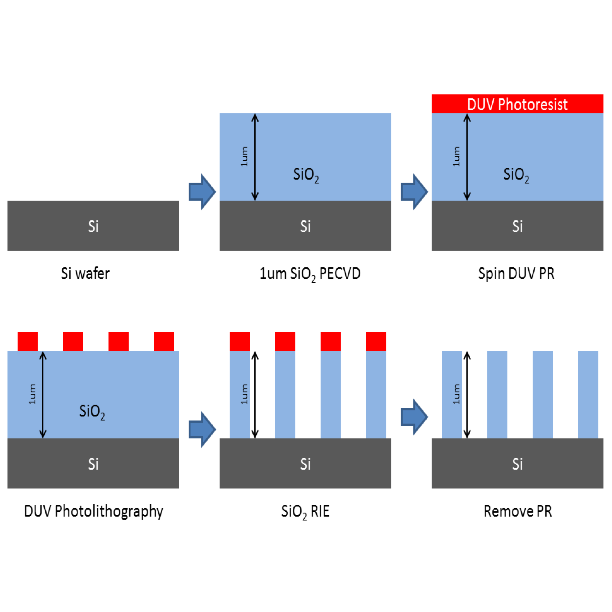
**Supporting Figure S1.** **Schematic of Microfabrication Procedure.** In this process, deep UV (DUV) lithography, plasma-enhanced chemical vapor deposition (PECVD), and reactive ion etching (RIE) were used. PR = photoresist.

### Electron Micrograph Acquisition

Scanning Electron Microscopy was performed with a Zeiss, LEO 1550 Field Emission Scanning Electron Microscopy (FE-SEM) to image the surface of the microfabricated mold.

### Data Analysis

### The thickness of and separation between protein-patterned regions were determined using image calibration and calipers in Metamorph (Molecular Devices). The percent of platelet area on the protein micropattern was determined using ImageJ. Measurements of platelet aspect ratio were determined by dividing the longest span of the platelet along the protein pattern by the longest span of the platelet perpendicular to the protein pattern using calipers in Metamorph. Percent of platelets spanning a region was determined by counting the number of platelets in contact with an edge that spanned a gap in protein and spread on two protein surfaces and then dividing this by the total number of platelets in contact with the edges of the two protein patches. Linear and exponential fits depicted in Figures 1 and 2 were created the built-in curve fitting functions of Igor (WaveMetrics).


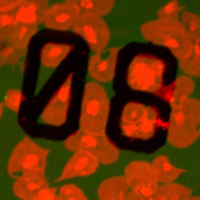


**Supporting Figure S2.** Images of platelets conforming to boundaries of the protein micropattern. Platelets are stained with CellMask (Invitrogen), a constitutive fluourecent membrane dye, and appear red. The micropatterns are comprised of fibrinogen-Alexa 488 (Invitrogen) and appear green. Scale bar denotes 5 μm.


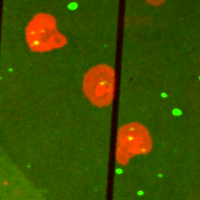


**Supporting Figure S3.** Additional image showing platelets conforming to the boundaries of the protein micropattern and adjust their spreading accordingly. Scale bar denotes 5 μm.


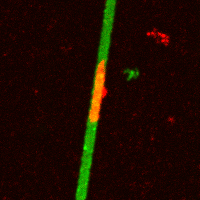


### **Supporting Figure S4.** On thinner micropatterned protein stripes (< 1μm), platelet spreading was more physically constrained and higher platelet aspect ratios were observed, with a maximum of 19. Scale bar denotes 5 μm.

# Supporting Video Captions

# Supporting Video S1. Geometrically constrained platelet spreading.

One platelet (left) lands on a BSA-blocked glass region near a narrow collagen micropatterned stripe. It then extends filopodia and moves onto the micropattern. While spreading, the platelet conforms to the boundaries of the protein and assumes a rectangular morphology. Meanwhile, another platelet (upper right) lands on a thinner protein stripe and exhibits similar behavior.

# Supporting Video S2. Platelet behavior at the protein-glass interface.

A platelet drifting past a fibrinogen micropattern lands on a region of BSA-blocked glass adjacent to the protein pattern. The platelet then extends filopodia and crawls over onto the protein region before fully spreading. As the distance of the non-patterned regions was >5 μm, the platelet could not span the micropatterned stripes, and spreading was confined to the protein micropattern. Elapsed time: 15 minutes, 50 seconds.

Supporting Videos S3 and S4. Platelet sensing using filopodia.

These videos show time lapsed responses of platelets as they come into contact with a microstamped protein surface. The platelets extend filopodia, which dynamically move around and either anchor to the protein or retract when not in contact with the protein. Lamellipodia then fill in the area between filopodia, and stay within the microstamped boundary. Elapsed Time for Video 3 is 22 minutes 20 seconds. Elapsed time for Video 4 is: 3 minutes, 10 seconds.

**References**

^1^Heemskerk JW, Hoyland J, Mason WT, Sage SO. Spiking in cytosolic calcium concentration in single fibrinogen-bound fura-2-loaded human platelets. *Biochem. J.* 1992;283(Pt 2): 379-383.

**^2^**Conde I, Cruz MA, Zhang H, Lopez JA, Afshar-Kharghan V. Platelet activation leads to activation and propagation of the complement system**.** *JEM.* 2005;201(6):871-879.
